# A single-center experience with 12 consecutive cases of pregnancy among patients with membranous ventricular septal aneurysm

**DOI:** 10.1186/s12884-017-1646-4

**Published:** 2018-01-03

**Authors:** Kana Wang, Xiaodong Wang, Haiyan Yu, Xinghui Liu, Aiyun Xing, Yong You

**Affiliations:** 1 Department of Obstetrics and Gynecology, West China Second University Hospital, Sichuan University, Chengdu, China; 2 Key Laboratory of Birth Defects and Related Diseases of Women and Children, (Sichuan University), Ministry of Education, Chengdu, China

**Keywords:** Membranous ventricular septal aneurysm (MVSA), Congenital heart disease, Pregnancy outcome

## Abstract

**Background:**

Membranous ventricular septal aneurysm (MVSA) is a rare cardiac anomaly that can occur as an isolated entity or being associated with other cardiac malformations. Complications of MVSA include thromboembolism, arrhythmia, rupture, bacterial endocarditis, right ventricular outflow tract obstruction, and atrioventricular valve diseases.The success rate of pregnancy and delivery in patients with MVSA has not been reported in the literature. This study was to assess the clinical implications of this condition from our center’s experience.

**Methods:**

This was a retrospective study for consecutive 12 pregnancies in women with MVSA, who delivered at a tertiary care center in west China between May 2008 and March 2015.

**Results:**

All patients with MVSA delivered via caesarian section. One patient with severe pulmonary arterial hypertension expired from pulmonary infection and heart failure after delivery. One patient terminated pregnancy in the second trimester- necessitated by cardiogenic shock. The other mothers had varying degrees of cardiac morbidity, but survived. Ten of thirteen newborns survived. Congenital heart disease and small-for-gestational-age (SGA) of newborn occurred in two cases (one twin and one single gestation). Two of these babies expired.

**Conclusions:**

Maternal and neonatal risk appeared associated with heart functional classifications, pulmonary hypertension and histories of cardiac events such as serious cardiac arrhythmia. Accurate diagnosis and care by a multidisciplinary team is recommended for pregnant woman with MVSA.

## Background

Membranous ventricular septal aneurysm (MVSA) is an uncommon anomaly of the membranous portion of the cardiac septum. Aneurysmal bulging begins in a weak or incomplete cardiac septum that is unable to overcome the differential pressure between the left and right ventricles. MVSA occurs as an isolated abnormality or being associated with other cardiac malformations. MVSA is a well-recognized risk factor for outflow tract obstruction and embolisms, and is related (either directly or secondary to) to rupture, bacterial endocarditis, and arrhythmia. However, most patients with this cardiac anomaly were asymptomatic, and the aneurysms were discovered incidentally on echocardiography. Without diagnosis, unforeseen problems or unexpected deaths may occur in patients who are under a conditions of increased cardiovascular stress, such as pregnancy. The outcome of pregnancy and delivery in patients with MVSA has not been reported in the literature. We present 12 cases of pregnant women with incidentally discovered MVSA and discussed our experience in managing these cases.

## Methods

This was a retrospective study approved by the Institutional Review Board of West China Second University Hospital. We reviewed the medical records of 591 pregnant women with congenital heart disease (CHD) who were admitted at West China Second University Hospital, a tertiary referral center, between May 2008 and March 2015. Prevalence of CHD in pregnancy was 0.88% (591/66988). Among 591 pregnant women with CHD, 12 cases of MVSA (2.03%) were diagnosed.

### Patient characteristics

The age of 12 pregnant women with MVSA ranged from 17 to 34 years old. All patients underwent clinical physical examination, electrocardiography (ECG), and transthoracic echocardiography. The co-morbidities of hypertension, diabetes mellitus, and other pregnancy associated significant conditions were also recorded and analyzed in the current study.

### Echocardiography

All included patients were evaluated by echocardiography. MVSA was diagnosed when the protrusion in the echocardiographic evaluation exceeded 7.5 mm [1]. The range of the protrusion, the mobility of the aneurismal structure, the size of the defect, left ventricular systolic function and the presence of other heart abnormalities were measured. Color flow Doppler was used to evaluate for the presence of septal shunt and valvular regurgitation, and to assess pulmonary artery pressure gradients. The left atrial/ventricular size or diameter was assessed in the transverse dimension in the parasternal longitudinal view (M-mode) and calculated planimetrically in the apical four-chamber view (2D). If mural thrombus was suspected, further echocardiographic evaluation was performed.

### Electrocardiogram

24-h ambulatory ECGs were performed in patients when they had abnormal electrocardiographic pattern; or in patients with abnormal electrolyte that might increase susceptibility to arrhythmia.

### Other examinations

MRI was used to confirm ischemic stroke. Vascular ultrasound was performed for patient with suspected peripheral embolism. Central venous catheterization (CVT) and/or right heart catheterization was undertaken to monitor central venous pressure (CVP) and pulmonary artery pressure gradients in patients who had signs of pulmonary hypertension.

## Results

### Cardiac complications

Eight cases of MVSA, heart disease was initially diagnosed during pregnancy. Five of these patients presented with chest tightness and shortness of breath at the second trimester. Three asymptomatic cases were found on the basis of systolic murmurs upon ausculation, and confirmed by echocardiography during the second trimester. In the remaining 4 cases (33.3%), diagnosis was made for the first time during labour. Cardiographic characteristics of the mothers are summarized in Table 1. Nine (75%) cases had cardiac function NYHA class III or higher. Five cases (41.7%) of MVSA existed in isolation, and 7 (58.3%) cases were complicated with ventricular septal defect.

**Table 1:** Characteristics of echocardiography and electrocardiogram in pregnant woman with MVSA

No.	Type of heart disease	LA/LV diameter (mm)	NYHA-FC	Range of protrusion (mm)	Size of the defect (mm)	PH^*^	Valve condition	Mural thrombus	LVSD^*^	Arrhythmia
1	VSA	Normal	II	13 × 13	No break	None	TR^*^	None	EF = 59%, FS = 31%	–
2	VSA VSD*	Normal	III	15 × 11	5	None	Normal	None	EF = 57%, FS = 30%	–
3	VSA VSD	Normal	III	16 × 15	11	Moderate	TR	None	EF = 71%, FS = 41%	Sinus tachycardia
4	VSA VSD	LA = 38, LV = 52	III	9 × 8	5	Mild	MR*	None	EF = 59%, FS = 33%	–
5	VSA VSD PDA*	Normal	III	17 × 12	5	Mild	MR + TR	None	EF = 60%, FS = 32%	Sinus tachycardia
6	VSA VSD	LA = 35, LV = 58	III	14 × 11	11	Moderate	Normal	None	EF = 58%, FS = 31%	Sinus rhythm with extreme right axis deviation (+85°)
7	VSA VSD	LA = 33, LV = 50	III	14 × 14	7	Moderate	TR	None	EF = 64%, FS = 34%	–
8	VSA	LA = 46, LV = 57	III-IV	28 × 14	No break	Mild	MR + TR	None	EF = 30%, FS = 14%	I°AVB, VPB
9	VSA VSD	LA = 38, LV = 57	IV	20 × 15	8	Severe	TR	None	EF = 59%, FS = 32%	IRBBB
10	VSA	Normal	II	12 × 12	4	None	Normal	None	EF = 71%, FS = 40%	Sinus tachycardia
11	VSA	Normal	III	16 × 10	No break	None	MR + TR	None	EF = 63%, FS = 34%	Sinus arrhythmia, APB
12	VSA	Normal	I–II	8 × 8	No break	None	Normal	None	EF = 69%, FS = 39%	–

*VSD* ventricular septal defect, *PDA* patent ductus arteriosus, *PFO* patent foramen ovale, *NYHA-FC* cardiac function grading (New York Heart Association), *PH* pulmonary hypertension, *LVSD* left ventricular systolic function, *LA/LV* Left atrial/ventricular, *MR* mitral regurgitation, *TR* tricuspid regurgitation, *AVB* atrioventricular block, *IRBBB* incomplete right bundle branch block, *VPB* ventricular premature beat, *APB* atrial premature beat

Upon diagnosis, the diameter of MVSA ranged from 8 mm to 28 mm (mean ± SD: 13.54 ± 4.31) and the protrusion area from 8 × 8 mm to 28 × 14 mm). Among them, 6 cases had a diameter greater than 15 mm. The aneurysm remained intact in 4 cases and was ruptured in 2 cases. Left cardiac diameter and left ventricular systolic function were assessed in all patients. Enlarged left heart chamber was found in 5 patients, and limited systolic function (EF = 30%, FS = 14%) was found in 1 patient.

In our study, the most common complications attributable to MVSA were pulmonary arterial hypertension (PAH) (58.3%), atrioventricular valve diseases (66.7%) and arrhythmias (58.3%). No thromboembolism was found in our cases. There were 3 cases (25%) of mild PAH, 3 cases (25%) of moderate PAH and 1 case (8.3%) of severe PAH. Eight patients had tricuspid and/or mitral regurgitation but none of them developed stenosis. According to ECG, one patient had 1st-degree ventricular premature beats (I AVB) and ventricular premature beats (VPB). One patient had atrial premature beats (APB); 3 patients had sinus tachycardia, and 1 patient had incomplete right bundle branch block.

### Obstetric complications

The clinical data of obstetric complications, maternal mortality and fetal/neonatal outcome are shown in Table 2. One case (NYHA class IV with severe pulmonary hypertension) had to terminate pregnancy at 17 weeks because of cardiogenic shock. She was then transferred to the Department of Cardiology for further treatments. Obstetric complications were found in 9 (81.8%) patients, including premature labor, premature rupture of membranes (PROM), preeclampsia, breech presentation, gestational diabetes mellitus (GDM), and postpartum hemorrhage resulting from twin pregnancy and placenta praevia. All cases had elective cesarean sections. Eight patients received combined spinal-epidural anesthesia, 1 epidural anesthesia, and 2 patients general anesthesia.

**Table 2:** Obstetrics characteristics of pregnancy complicated with MVSA

No.	Age	BMI	Gravidity and parity history	Obstetric complication	Mode and time of delivery	Anesthesia methods	Apgar Score (1–5-10 min)	Outcomes	HOD (day)	Antibiotics and day uses
1	29	26.56	G1P0	None	Caesarean section: 38 + 2 weeks	Combined spinal epidural	10–10-10	M – good N – live, 3175 g	6	Ceftezole (5d)
2	17	25.08	G1P0	Prematurity, Preeclampsia Breech presentation	Caesarean section: 31 + 1 weeks	Epidural anesthesia	10–10-10	M – good N – live, 2050 g,with PDA and PFO	9	Cefoxitin (2.5d)
3	32	27.04	G3P1	GDM	Caesarean section: 37 weeks	General anesthesia	10–10-10	M – good N – live, 3250 g	6	Cefoxitin (5d)
4	33	24.22	G2P0	Prematurity, PROM	Caesarean section: 34 + 1 weeks	Combined spinal epidural	9–10-10	M – good N – live, 2200 g	12	Cefmetazole (12d)
5	24	23.37	G2P0	None	Caesarean section: 39 + 2 weeks	Combined spinal epidural	10–10-10	M – good N – live, 3600 g	4	Cefoxitin (2d)
6	21	22.60	G1P0	Prematurity, Moderate anemia	Caesarean section: 33 + 6 weeks	General anesthesia	8–9-10	M – good N – live, 2180 g	10	Cefoxitin (7d)
7	19	19.56	G1P0	Prematurity	Caesarean section: 34 weeks	Combined spinal epidural	8–9-10	M – good N – live, 2260 g	8	Piperacillin- tazobactam (7d)
8	25	18.43	G1P0	None	Terminate pregnancy: 17 weeks	Combined spinal epidural	None	M – Cardiac shock, transferred to the Department of Cardiology N – died, 80 g	3	Ceftriaxone(2d)
9	19	20.30	G1P0	Prematurity	Caesarean section: 30 + 4 weeks	General anesthesia	4–5-8	M – died with pulmonary infection and heart failure N – live, 1095 g, SGA, died in the following day	8	Cefoxitin (2d) Meropene (5d)
10	29	25.20	G2P0	Prematurity, PROM	Caesarean section: 36 + 5 weeks	Combined spinal epidural	10–10-10	M – good N – live,2930 g	3	Cefoxitin (2d)
11	30	23.44	G5P1	Prematurity, MCDA, Placenta praevia, Postpartum hemorrhage	Caesarean section: 34 + 4 weeks	Combined spinal epidural	N1: 9–10-10	M – good, with pulmonary infection N (twins) – 1: live, 1550 g, with SGA; 2: died, 2080 g, with TOF	46	Meropene (14d) Piperacillin tazobactam (7d)
12	34	26.53	G6P1	Breech presentation	Caesarean section: 38 + 5 weeks	Combined spinal epidural	10–10-10	M – good N – live, 2800 g	6	Cefoxitin (2d)

*VSD* ventricular septal defect, *PDA* patent ductus arteriosus, *BMI* body mass index (kg/cm^2^), *PROM* premature rupture of membranes, *GDM* gestational diabetes mellitus, *MCDA* monochorionic diamnionic twin, *M* maternal, *N* neonate, *PFO* patent foramen ovale, *TOF* Tetalogy of Fallot, *HOD* hospital day (total days in hospital)

### Maternal and perinatal outcomes

In this study, 6 (50%) woman were primigravid (Table 2). The case fatality rate in the study was 8.3% with death resulting from pulmonary infection and heart failure. Most neonates, 10 (76.9%) including a twin pregnancy had birth weights more than 2000 g but less than 2500 g. Three neonates (38.5%) weighed more than 2500 g. Neonatal case fatality rate was 15.4%. Congenital heart disease was noted in 2 babies (15.4%), one with TOF, and the other with PDA and PFO. The rate of small-for-gestational-age (SGA) (estimated infant weight after delivery below the 10th percentile for corresponding gestational age) was 15.4%.

Of note, maternal and neonatal mortality occurred only in patient with a MVSA protrusion diameter > 15 mm and NYHA class III-IV. The maternal cardiac anomaly and the outcome of pregnancies are summarized in Table 3.

**Table 3:** Outcome of pregnancies with different maternal cardiac anomaly

Parameter of anomaly	Number.of case (Fetal/maternal)	Prematurity	SGA	Fetal death	Neonatal death	Maternal death
Total, n (%)	13/12	7 (58.3%)	2 (15.4%)	1 (7.7%)	2 (15.4%)	1 (8.3%)
Size of VSA						
< 10 mm	2/2	1 (50%)	0	0	0	0
10–15 mm	5/5	4 (80%)	0	0	0	0
> 15 mm	6/5	2 (40%)	2 (33.3%)	1 (16.7%)	2 (33.3%)	1 (16.7%)
Defect of VSA						
Intact	5/4	1 (25%)	1 (20%)	1 (20%)	1 (20%)	0
≤ 5 mm	4/4	3 (75%)	0	0	0	0
> 5 mm	4/4	3 (75%)	1 (25%)	0	1 (25%)	1 (25%)
LVSD						
Normal	12/11	7 (63.6%)	2 (16.7%)	0	2 (16.7%)	1 (8.3%)
Dysfunction	1/1	0	0	1 (100%)	0	0
NYHA-FC						
I–II	3/3	1 (33.3%)	0	0	0	0
III–IV	10/ 9	6 (66.7%)	2 (20%)	1 (10%)	2 (20%)	1 (10%)
PH						
None	6/5	3 (60%)	1 (16.7%)	0	1 (16.7%)	0
Mild	3/3	1 (33.3%)	0	1 (33.3%)	0	0
Moderate	3/3	2 (66.7%)	0	0	0	0
Severe	1/1	1 (100%)	1 (100%)	0	1 (100%)	1 (100%)
Congenital abnormality						
None	5/4	2 (50%)	1 (20%)	1 (20%)	1 (20%)	0
VSD	7/7	5 (71.4%)	1 (14.3%)	0	1 (14.3%)	1 (14.3%)
PDA	1/1	0	0	0	0	0
Valve condition						
Normal	4/4	3 (75%)	0	0	0	0
TR	4/4	2 (50%)	1 (25%)	0	1 (25%)	1 (25%)
MR	1/1	1 (100%)	0	0	0	0
TR + MR	4/3	1 (33.3%)	1 (25%)	1 (25%)	1 (25%)	0
Arrhythmia						
None l	5/5	3 (60%)	0	0	0	0
Sinus tachycardia	4/4	2 (50%)	0	0	0	0
I°AVB	1/1	0	0	1 (100%)	0	0
IRBBB	1/1	1 (100%)	1 (100%)	0	1 (100%)	1 (100%)
Sinus tachycardia + APB	2/1	1 (100%)	1 (50%)	0	1 (50%)	0

*NYHA-FC* cardiac function grading (New York Heart Association), *PH* pulmonary hypertension, *VSD* ventricular septal defect, *PDA* patent ductus arteriosus, *TR* tricuspid regurgitation, *MR* mitral regurgitation, *AVB* atrioventricular block, *IRBBB* incomplete right bundle branch block, *SGA* Small for gestational age

## Discussion

Ventricular septal aneurysms are extremely rare clinicopathological entities that include congenital muscular interventricular septal aneurysm (CMISA) and membranous ventricular septal aneurysm (MVSA) [2, 3]. Membranous ventricular septal aneurysm was first described as a congenital malformation in 1826 by Laennec [4]. It is thought that the membranous septum remains weak during embryonic development, and forms an aneurysm of the membranous septum—bulging toward the right under the pressure of left ventricle. It has also been proposed that MVSA could be acquired—the consequence of delayed natural closure of a ventricular septal defect after birth [5–7]. Indeed, MVSA is primarily associated with a history of spontaneous closure of a small membranous ventricular septal defect in childhood [2]. Patients with MVSA may incur potential cardiac complications, such as aortic valve prolapse, right ventricular outflow obstruction, tricuspid valve insufficiency, arrhythmia, rupture, thromboembolism, and bacterial endocarditis [8–12]. However, most MVSA patients are asymptomatic, the physical examination and electrocardiogram may fail to identify MVSA in the absence of ventricular arrhythmia or other complications [13]. MVSA may provide an occult cardiac source of cerebral or systemic embolus [14]. Furthermore, congestive heart failure may occur because of adverse effects of MVSA on systolic function [9].

Our subjects were asymptomatic before conception, or experienced mild symptoms, and physician did not pay enough attention to them. During pregnancy, the most common complications in our cases were pulmonary arterial hypertension, atrioventricular valve diseases and arrhythmia. No incidence of thromboembolism was found, and this may have assisted our clinical outcomes, which were basically good in spite of varied degrees of pulmonary arterial hypertension in 7 patients. The numbers of patients in the subgroups were too small to conduct further statistical analysis.

Asymptomatic patients with MVSA should be followed closely for potential cardiac complications. This is especially important for the cases in the setting of pregnancy [15–17]. Pregnancy places extra load on the heart, and can affect cardiac function, leading to increased maternal morbidity and perinatal morbidity [18, 19]. In general, women with NYHA class > III have a relatively poor prognosis during pregnancy [20]. In our study, one case with NYHA class IV and severe pulmonary hypertension had to terminate pregnancy at 17 weeks of gestation; and one case with NYHA class IV expired.

We assumed that pregnant women with MVSA were at risk for maternal and neonatal complications, although the outcome in pregnancy with MVSA has not been reported in the literature. There was indeed a high rate (> 80%) of obstetric events involving prematurity, PROM, preeclampsia, breech presentation, GDM and postpartum hemorrhage. Mothers with CHD also have a high incidence of fetal complications such as miscarriage, premature births, low birth weights, respiratory distress syndrome, and cardiac anomalies [21]. In this study, neonatal congenital heart disease (Tetralogy of Fallot) and SGA at birth (1550 g at 34 + 4 weeks, survived) occurred in a twin gestation, the former expired and the latter survived. An additional singleton baby was born with SGA (1095 g at 30 + 4 weeks gestation) and expired on day 2 post birth.

Due to high risk of complications during vaginal delivery, a cesarean section delivery is usually recommended for woman with cardiac disease, since extra cardiac burden from prolonged laboring process may be avoided [17, 22]. In the current study, except one terminated pregnancy in the second trimester, all of the remaining patients delivered by cesarean section under combined spinal epidural anesthesia, epidural anesthesia or general anesthesia. Except one maternal death from pulmonary infection and heart failure (also with severe pulmonary arterial hypertension, NYHA class IV), all other patients survived. Almost all of them suffered cardiac complications such as mild-to-moderate pulmonary arterial hypertension, atrioventricular valve diseases and arrhythmia, but none of them developed thromboembolism. The relative good outcomes in our patients may be at least partially owing to the absence of severe hypertension, cardiac failure and cerebral and pulmonary embolism. In addition, comprehensive interdisciplinary management among the cardiologist, obstetrician, anesthetist, and neonatologist, and detailed plans for delivery may have improved the prognosis [23].

## Conclusion

Pregnancy with MVSA presents an increased risk of cardiac complications. Maternal and neonatal risks seem to be associated with heart functional classification, pulmonary hypertension and history of serious cardiac arrhythmia. Accurate diagnosis and care by a multidisciplinary team should be the best approach in order to improve prognosis in these patients.

## Abbreviations

AVB: Atrioventricular block
CHD: Congenital heart disease
GDM: Gestational diabetes mellitus
IRBBB: Incomplete right bundle branch block
MVSA: Membranous ventricular septal aneurysm
NYHA-FC: Cardiac function grading (New York Heart Association)
PDA: Patent ductus arteriosus
PFO: Patent foramen ovale
PH: Pulmonary hypertension
PROM: Premature rupture of membranes
SGA: Small for gestational age
VPB: Ventricular premature beat
VSD: Ventricular septal defect
